# Efficacy of Ciprofloxacin, Metronidazole and Minocycline in Ordered Mesoporous Silica against *Enterococcus faecalis* for Dental Pulp Revascularization: An In-Vitro Study

**DOI:** 10.3390/ma15062266

**Published:** 2022-03-18

**Authors:** Cintia Micaela Chamorro-Petronacci, Beatriz Santos Torres, Rocío Guerrero-Nieves, Mario Pérez-Sayáns, Marcia Carvalho-de Abreu Fantini, Luis Carlos Cides-da-Silva, Beatriz Magariños, Berta Rivas-Mundiña

**Affiliations:** 1 ORALRES Group, Department of Oral Medicine, Instituto de Investigación Sanitaria de Santiago de Compostela (IDIS), Travesía da Choupana S/N, 15706 Santiago de Compostela, Spain; perezsayans@gmail.com; 2 Department of Microbiology and Parasitology, CIBUS-Faculty of Biology, University of Santiago de Compostela, Campus Vida, 15705 Santiago de Compostela, Spain; bs.torres@usc.es (B.S.T.); beatriz.magarinos@usc.es (B.M.); 3 MedOralRes Group, Department of Surgery and Medical-Surgical Specialities, Universidade de Santiago de Compostela (USC), Entrerrios S/N, 15705 Santiago de Compostela, Spain; rocio.guerrero.nieves@gmail.com (R.G.-N.); berta.rivas@usc.es (B.R.-M.); 4 Institute of Physics, Universidade de São Paulo, Rua do Matão, Sao Paulo 05508-090, SP, Brazil; mfantini@if.usp.br (M.C.-d.A.F.); luiscides@gmail.com (L.C.C.-d.-S.)

**Keywords:** antibiotics, *Enterococcus faecalis*, microbial sensitivity tests, pulp revascularization

## Abstract

Pulp revascularization of teeth with necrotic pulp has become an alternative treatment in cases with immature apex. Microbial control is essential to achieve a successful outcome and continued root development. *Enterococcus faecalis* (*E. faecalis*) is the most frequently isolated bacterial species in root canals of endodontically failed teeth. Our main goal was to compare the in-vitro antimicrobial efficacy of different antibiotic formulations delivered by ordered mesoporous silica (OMS) against *E. faecalis*. To determine antibiotic susceptibility, we tested OMS and triple antibiotic paste (TAP; ciprofloxacin:metronidazole:minocycline) with different reagents in different concentrations, using the Kirby–Bauer disk diffusion method. OMS and metronidazole showed no antibacterial activity against *E. faecalis*. Mixtures of OMS and antibiotics in proportions of 2:2:14 and 4:1:7 (mg/L of ciprofloxacin:metronidazole:minocycline, respectively) showed the lowest antibacterial activity. The antibacterial activity of the combined solutions of ciprofloxacin and metronidazole was significantly higher (*p* < 0.005). Combinations in different concentrations of minocycline, ciprofloxacin, and metronidazole in OMS have shown activity against *E. faecalis*, although the combined use of ciprofloxacin and metronidazole has shown the most effective results. This study demonstrates the efficacy of intracanal antibiotic combination paste activity against *E. faecalis,* avoiding the use of minocycline, whose undesirable effect of teeth staining is a common problem for patients and professionals in dental clinic.

## 1. Introduction

Traumatic dental injury in young patients is a common occurrence [1] and some cases finally result in dental pulp necrosis. Any damage to the pulp tissue of the tooth can stop root development, resulting in an open apex; an altered crown-root relationship; thin dentin walls; and, consequently, a tooth more susceptible to fracture [2]. Therefore, depending on the stage of root development, treatment options are conventional endodontics, apexification or apicoformation, and pulp revascularization [3]. Pulp revascularization is a regenerative procedure of the dentin-pulp complex that aims to achieve apical closure in immature permanent teeth with pulp necrosis.

The elimination of bacteria from the root canal system plays an important and a critical role in the success of endodontic treatment [4]. Considering the anatomical complexity of the root canal space, mechanical instrumentation alone has been shown to be insufficient to provide the proper environment [5]; moreover, in immature teeth it can cause a further weakening of the root dentinal walls [6].

Traditionally, calcium hydroxide (Ca(OH)^2^) was used for apexification with mineral trioxide aggregate (MTA) to induce hard tissue apical barriers in incompletely formed root in which the pulp is diagnosed as necrosis [7]. This procedure involved several appointments in which the Ca(OH)_2_ had to be removed until the root canal could be sealed by conventional techniques with gutta-percha with MTA apical stopper. Nevertheless, according to Khoshkhounejad et al., cell viability is significantly reduced when using high concentrations of Ca(OH)^2^ intracanal paste (*p* > 0.05), due to its high cytotoxicity [8]. It is important to maintain a balance between the antibacterial efficacy of chemical reagents and their harmlessness to stem cells.

Pulp revascularization procedure emerges as a more biological and conservative alternative to the apexification technique and is defined as a procedure based on biology, designed to physiologically replace damaged tooth structures, as well as the cells of the pulpo-dentin complex [9].

The application of intracanal medication for pulp revascularization is essential to eliminate endodontic pathogens and, considering the polymicrobial nature of dental infections, a combination of antibiotics reduces possible bacteria resistance [10]. Due to its broad-spectrum antimicrobial activity, Triple Antibiotic Paste (TAP) has been widely used as a combination of metronidazole, ciprofloxacin, and minocycline [11].

Recent meta-analyses have revealed that both treatments, apexification and revascularization, are effective options for the treatment of apical periodontitis and apex closure; however, pulp revascularization is more effective in achieving elongation and increased root thickness [3]. Likewise, the efficacy of TAP can be improved with the use of nanotechnology, using antibiotic polymeric nanofibers to deliver the antibiotic concentration in the apex zone for the regeneration of pulp and dentin procedure [12].

Ordered mesoporous silica (OMS) is a promising alternative for difficult-to-access bacterial infections and excellent candidates for developing specific devices for controlled intra-canal medication delivery, due to their high biocompatibility, high drug-loading capacity, and versatility of chemical surface modification. Their orderly pore structure and stable physicochemical properties make them widely used as pharmacological carriers.

Moreover, OMS has been demonstrated to significantly accumulate and infiltrate in root canal system, which may provide a potential approach for further applying encapsulated antimicrobials in advanced endodontic therapy [13]. However, results are scarce and preliminary [14]; for this reason our research team has developed new antibiotic loaded OMS. TAP has also demonstrated efficacy against *E. faecalis,* but there is a lack of evidence regarding the effective concentration to be used for the procedure [15] since there are contradictory results in the literature [16].

Our main goal was to compare the in vitro antimicrobial efficacy of different antibiotic formulations delivered by OMS against *E. faecalis*.

## 2. Materials and Methods

*E. faecalis* was routinely cultured in Trypticase Soy Agar (TSA) (Condalab, Madrid, Spain) and incubated at 37 °C for 24 h. The *E. faecalis* (ATCC 19433TM) strain from the American Type Culture Collection (ATCC, Manassas, VA, USA) was used (Thermo Scientific™ de *Enterococcus faecalis* ATCC™ 19433™, Manassas, VA, USA).

### 2.1. Solution Preparation

The different solutions used for this study were prepared at the Galicia Ceramic Institute in Santiago de Compostela (Spain) by dissolving different amounts of antibiotic powder in distilled water according to the following manufactured antibiotic: 100 mg Minocyclin (Genfar, Colombia INVIMA 2008M-0007991; R.S. EE-03106), 500 mg Ciprofloxacin (Laboratorios Natualres y Genéricos, S.A.C., R.S. EN-05139), and 500 mg Metronidazol (Laboratorios Naturales y Genéricos, S.A.C., R.S. EN-05089). Table 1 and Table 2 show the characteristics of the eight solutions with the different concentrations of antibiotics, as well as the ordered mesoporous silica SBA-15 (Santa Barbara Amorphous-15) (University of California, Santa Barbara, CA, USA), synthesized as reported by Zhao et al. [17].

### 2.2. Antimicrobial Assessment

The antimicrobial effects of different solutions were tested against *E. faecalis* grown on TSA plates for 24 h.

Susceptibility to different antibiotics was determined using the Kirby–Bauer disk diffusion test on MHA (Thermo Scientific™ Oxoid™, Hants, UK) [18], using the following chemotherapeutic agents (20 μL per disk): ciprofloxacin (C, 500 mg/L), metronidazole (Me, 500 mg/L), and minocycline (Mi, 100 mg/L). To determine the efficacy of each antibacterial compound, the inhibition halos observed around the antibiotic loaded disk were measured.

Bacteria were suspended with a sterile swab in 2 mL of saline solution and adjusted to a turbidity of 0.5 on the McFarland scale (equivalent to 1 × 10^8^–2 × 10^8^ CFU/mL of *E. coli*) [19]. Subsequently, the bacteria were seeded following the MHA plate seeding method. The discs impregnated with 20 µL of each corresponding antibiotic solution were then deposited. Table 3 specifies the tests performed in this study.

Plates were incubated for 24 h at 37 °C to later analyse the results. After said time, the results obtained from the bacterial activity were measured using the diameter in millimetres (mm) with a calliper, thus obtaining the size of the inhibition halos.

### 2.3. Minimal Inhibitory Concentration (MIC)

MIC values were determined to define the antimicrobial efficacy of the antibiotics studied. *E. faecalis* ATCC 19433 ™ was cultivated using TSB medium (Thermo Scientific™ Remel™ Tryptic Soy Broth, Manassas, VA, USA), to determine the MIC of each compound and its combinations following the microdilution protocol described by Wiegand et al. [20]. The following concentrations were tested: (1) mixture of Ciprofloxacin (4 mg/L), Metronidazole (4 mg/L), and Minocycline (5.6 mg/L); (2) mixture of Ciprofloxacin (2 mg/L) and Metronidazole (2 mg/L).

### 2.4. Statistical Analysis

Statistical analyses were performed by using SPSS statistical software 22.0 for Windows. Descriptive analysis was used with media and standard deviation to quantitative variables The effect of antibacterial activity against *E. faecalis* and the halo inhibition diameter was quantified using the Student’s t test. Significance was set at *p* < 0.05.

## 3. Results

A total of 8 different formulations were tested to assess antimicrobial effectiveness. The results of the inhibition halos are summarized in Table 4. The mean halo diameter was 11 mm (SD = 7.81, range 0–21).

**Figure 1:**
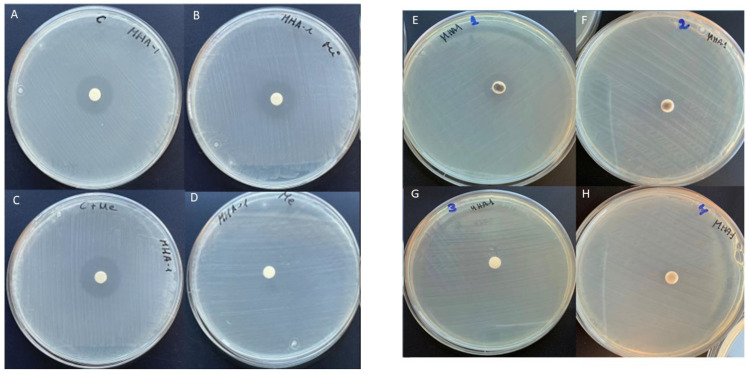
Halos of inhibition against *E. faecalis*. (**A**) Ciprofloxacin; (**B**) minocycline; (**C**) ciprofloxacin + metronidazole. (**D**) metronidazole; (**E**) SBA-15 + 4:4:28 *, SBA-15 (Santa Barbara Amorphous 15) OMS (Ordered Mesoporous Silica); (**F**) SBA-15 + 2:2:14 *; (**G**) SBA-15; and (**H**) SBA-15 + 1:1:17 *. * (Ci:Me:Mi).

**Figure 2:**
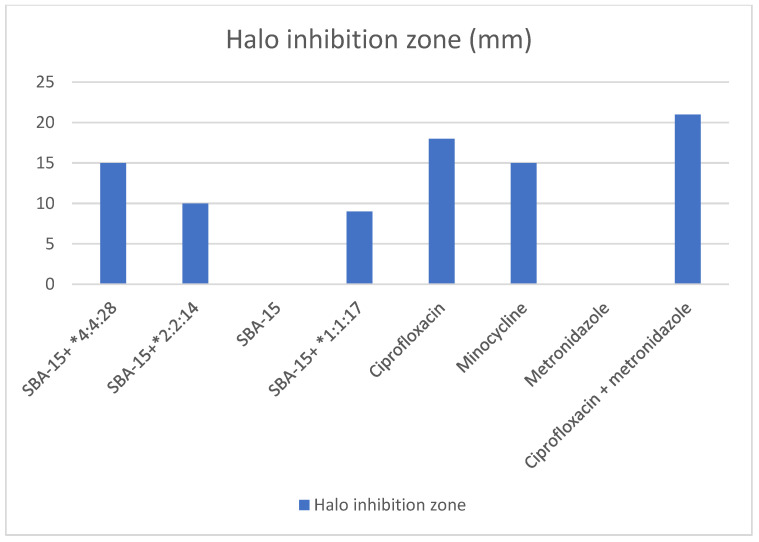
Antibacterial activity of the different drug solutions evaluated. * (Ci:Me:Mi).

The antibacterial activity has been assessed with halo diameter inhibition and is summarized in Figure 1. The greatest antibacterial effectiveness was demonstrated by the combination of ciprofloxacin with metronidazole (*p* < 0.005), observing an inhibitory zone of 21 mm (Figure 2). Plates containing exclusively SBA-15 (with a concentration of 100 mg/L) and the metronidazole plate (with a concentration of 500 mg/L) had no antimicrobial effect. All the other tested solutions resulted in some degree of inhibitory effect (Figure 1).

**Figure 3:**
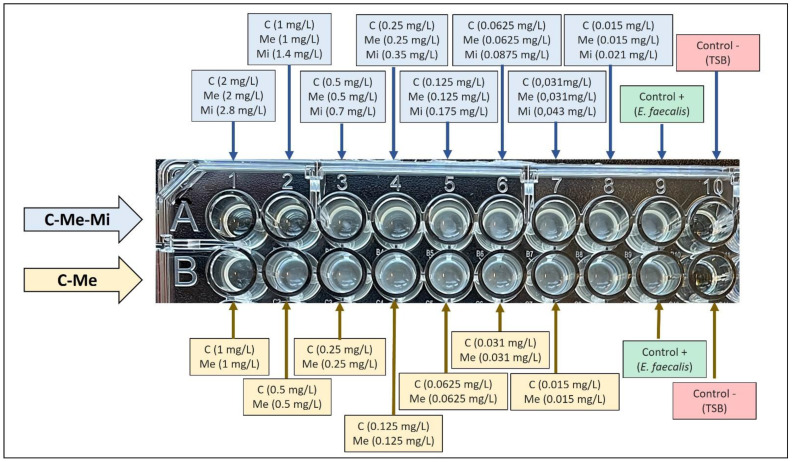
MIC determination of different antibiotic concentrations against *E. faecalis*; C (ciprofloxacin), Me (metronidazole), and Mi (minocycline); mg/L (milligrams per liter); TSB (Trypticase Soy Broth).

MIC results (Figure 3) revealed that *E. faecalis* is sensitive to the concentrations of the mixture of ciprofloxacin (2 mg/L), metronidazole (2 mg/L), minocycline (2.8 mg/L), ciprofloxacin (1 mg/L), metronidazole (1 mg/L), and minocycline (1.4 mg/L), e.g., half of the normally used concentration. Regarding the ciprofloxacin and metronidazole mixture, *E. faecalis* is sensitive to the concentration of ciprofloxacin (1 mg/L) and metronidazole (1 mg/L), again, half the normally used concentration. However, this pathogen is not sensitive to lower concentrations of ciprofloxacin (1 mg/L), metronidazole (1 mg/L), minocycline (1.4 mg/L), ciprofloxacin (1 mg/L), and metronidazole (1 mg/L).

## 4. Discussion

The surface area and pore volume after encapsulation of the antibiotics decreased around 40%, Therefore, there are free pores to encapsulate high concentrations of antibiotics, if necessary, or to decrease the silica mass in the medication formula for in-vivo use. *E. faecalis* is a Gram-positive, immobile, facultative anaerobic pathogen that acts as an opportunistic microorganism [21] in different oral diseases, including endodontic infections [22]. The presence of *E. faecalis* in the dental root canals reduces the success rate of endodontic treatments and is considered one of the main reasons for endodontic failure and the persistence of periapical infection [23]. To eliminate the microorganisms from the root canal system in pulp revascularization treatment, different solutions are used, including sodium hypochlorite and TAP, whose wide spectrum of action and disinfection capacity have been widely demonstrated [24].

We have investigated the activity of each of the compounds in TAP separately for *E. faecalis* growth inhibition to know if the results obtained are due to the mixture of the antibiotics with different concentrations or due to one of its components instead. The findings of our study indicated that the metronidazole and ciprofloxacin combination has greater antibacterial efficacy against *E. faecalis* compared to the other antibiotics and combinations with OMS.

Metronidazole is a broad-spectrum antibacterial drug used against Gram-positive anaerobic bacteria such as *E. faecalis* (10). However, our results showed a null efficacy against growth inhibition, similar to the findings by Bottino et al., who studied the effect aga *E. faecalis* and *P. gingivalis* [25].

Ciprofloxacin is a bactericidal antibiotic with high effectivity against Gram-negative bacteria but limited against Gram-positive bacteria and most anaerobic bacteria. This antibiotic has been found to have the highest activity against *E. faecalis* when it is compared alone to other antibiotics. Our findings agree with previous studies where the use of antibiotic-releasing nanofibers of ciprofloxacin and metronidazole showed efficacy against *E. faecalis*, *P. gingivalis*, and *Fusobacterium nucleatum*, also reducing the impact on cell viability [26].

Minocycline is a broad-spectrum antimicrobial and bacteriostatic tetracycline against most anaerobic and facultative bacteria, and gram-positive and gram-negative microorganisms [10]. It invades bacterial cells by passive diffusion through the outer membrane and by active transport through the inner membrane, reaching ribosome surfaces and inhibiting bacterial protein synthesis. The main limitation is its binding capacity for ions by chelation and the development of insoluble complexes, thus increasing substantivity and hindering angiogenesis and regeneration [12].

The best results in our study were obtained when using ciprofloxacin combined with metronidazole, demonstrating a synergistic effect, as previously observed by other researchers who reported increased effectivity against *E. faecalis* [27]. This solves a common concern among clinicians who use TAP, since minocycline can be now omitted to avoid dentin staining [28,29]. Minocycline is a broad-spectrum antibiotic, effective against Gram-positive microorganisms [11], whose high antimicrobial activity against *E. faecalis* has also been demonstrated in this study. However, as mentioned above, we can achieve better results with the combination of metronidazole and ciprofloxacin, avoiding the dreaded side effect of permanent staining due to minocycline.

Traditionally, TAP clinical concentration used was approximately 1000 mg/mL, however, it has been demonstrated that this concentration can avoid the apical stem cell survival [28]. The current clinical recommendation by the American Endodontic Association suggests the use of 100 mg/L TAP because this concentration of TAP had no cytotoxic effect on dental pulp stem cells [30]. However, the antibacterial effect was not demonstrated to be enough to completely eradicate the biofilm of *E. faecalis*. The results obtained in this study indicate that, regarding our MIC assays, lower concentrations of ciprofloxacin at 1 mg/L combined with metronidazole 1 mg/L and with minocycline 1.4 mg/L, as well as ciprofloxacin 1 mg/L combined with metronidazole 1 mg/L, have no antibiotic effect against *E. faecalis*.

Different proportions and concentrations of TAP has been tested among literature reviewed. Cunha-Neto et al. have used 1: 1: 1 ratio of antibiotics and concentration of 5 mg/mL, which means 5000 mg/L, resulting in an excess, as we have already explained for endodontic regeneration therapy [31]. Other reviewed articles also use TAP with 1:1:1 ratio combination, with some explaining concentrations and others not [32,33]. We have used commercial preparations (see Material and Method section) for antibiotic samples, and we wanted to have the same concentration for all of them; for this reason, we have used different combination in a proportional way to determine the efficacy.

The low antibacterial capacity of OMS in infected areas demonstrated by Fan et al. [14] agree with our findings, as OMS alone show no antibacterial activity as metronidazole did. Silicate mesoporous has demonstrated the ability to promote mineralization and release of molecules in different conditions, without affecting cell proliferation. Other formulations have been proved, such as chlorhexidine, to evaluate its effect on mineral release and antibacterial activity affected in different media conditions. Although the chemical mechanism still needs further investigation, type of bioactive molecule, pH, and the existence of organic components seemed to affect the efficacy [13]. We have expected to obtain more respectful and controlled local administration through OMS releasing antibiotics in the root canal [34], although it has no effect against *E. faecalis*.

Limitations to our study include the limited sample used, the assessment of the cytotoxicity of the different antibiotic formulations, and their concentrations with primary human gingival fibroblast cell lines [35]. The next interesting research could be to compare our results with other physical effective techniques as photodynamic therapies [35]. Although previous works have demonstrated the efficacy of antibiotic combinations without OMS [5], an interesting step to compare results has been to prove this combination in our lab before OMS loading. Another limitation of this study is the pH medium effect on OMS antibiotic realization, which could limit the antibacterial defect. On the other hand, with the results obtained, this analysis can be carried out with the concentrations and antibiotics that we have shown to be effective. Furthermore, we cannot forget the polymicrobial nature of endodontic infections, as well as the remaining tissues and fluids in the root canal system that can reduce the efficiency of intracanal drugs, which would require an additional in vitro model.

## 5. Conclusions

Combinations at different concentrations of minocycline, ciprofloxacin, and metronidazole in mesoporous silica have shown activity against the bacterium *E. faecalis*. The combined use of ciprofloxacin and metronidazole has revealed the most effective results against *E. faecalis*. Both metronidazole and SM used in isolation have shown a null effect against this pathogen.

## Figures and Tables

**Table 1:** Physical characteristics of the used concentrations.

Dissolution	Milligrams (mg)	Volume (mL)	Concentration (mg/L)
Ciprofloxacin (Ci)	10.0	20	500
Metronidazole (Me)	10.0	20	500
Minocycline (Mi)	1.8	18	100
Ci + Me	7.0 7.1	14 14	500 507

**Table 2:** Solutions tested in the study. * SBA-15 (Santa Barbara Amorphous 15, OMS) ordered mesoporous silica (OMS). ** (Ci:Me:Mi). The surface area, total pore’s volume, and mean pore diameter were determined using ASAP 2020-Micromeritics^®^ (ATS Scientific Inc., Burlington, ON, Canada) porosimeter in nitrogen atmosphere.

Sample (mL:mL:mL)	S_BET_ (m^2^/g)	Total Volume (cm^3^/g)	Mean Pore Diameter (nm)
SBA-15 * (100 mg/L)	968	2.16	10.2 ± 0.1
1:1:7 **	628	1.59	10.2 ± 0.1
2:2:14 **	647	1.72	10.2 ± 0.1
4:4:28 **	530	1.47	10.1 ± 0.1

**Table 3:** Numbering and nomenclature of each plate with its corresponding solution. * SBA-15 (Santa Barbara Amorphous 15, Orderer Mesoporous Silica, OMS).

PLATE	Solution
1	SBA-15 + 4:4:28 (Ci:Me:Mi)
2	SBA-15 + 2:2:14 (Ci:Me:Mi)
3	SBA-15 100 mg/mL
4	SBA-15 + 1:1:7 (Ci:Me:Mi)
C	Ciprofloxacin
Mi	Minocycline
Me	Metronidazole
C + Me	Ciprofloxacin and metronidazole

**Table 4:** Bacterial Inhibition Halos (mm). * (Ci:Me:Mi). SBA-15 (Santaba Barbara Amorphous 15, Ordered mesoporous silica, OMS).

Plate	Halo Diameter (mm)
A (C)	18
B (Mi)	15
C (C + Me)	21
D (Me)	0
E (SBA-15 + 4:4:28 *)	15
F (SBA-15 + 2:2:14 *)	10
G (SBA-15)	0
H (SBA-15 + 1:1:17 *)	9

## Data Availability

Not applicable.
